# The Effect of Nb Doping on the Properties of Ti-Al Intermetallic Compounds Using First-Principles Calculations

**DOI:** 10.3390/ma17020358

**Published:** 2024-01-11

**Authors:** Kun Wang, Hongping Xiang, Lin Xu, Aihan Feng, Shoujiang Qu, Hao Wang, Daolun Chen

**Affiliations:** 1 Shanghai Key Laboratory of D&A for Metal-Functional Materials, School of Materials Science & Engineering, Tongji University, Shanghai 201804, China; 2032921@tongji.edu.cn (K.W.); xianghp@tongji.edu.cn (H.X.); qushoujiang@tongji.edu.cn (S.Q.); 2 Biomaterials R&D Center, Zhuhai Institute of Advanced Technology, Chinese Academy of Sciences, Zhuhai 519000, China; linxu19802022@163.com; 3 Interdisciplinary Center for Additive Manufacturing, School of Materials and Chemistry, University of Shanghai for Science and Technology, Shanghai 200093, China; haowang7@usst.edu.cn; 4 Department of Mechanical, Industrial and Mechatronics Engineering, Toronto Metropolitan University, Toronto, ON M5B 2K3, Canada

**Keywords:** Ti-Al compounds, first-principles theory, mechanical properties, electronic structures

## Abstract

The crystal structures, stability, mechanical properties and electronic structures of Nb-free and Nb-doped Ti-Al intermetallic compounds were investigated via first-principles calculations. Seven components and eleven crystal configurations were considered based on the phase diagram. The calculated results demonstrate that hP8-Ti_3_Al, tP4-TiAl, tP32-Ti_3_Al_5_, tI24-TiAl_2_, tI16-Ti_5_Al_11_, tI24-Ti_2_Al_5_, and tI32-TiAl_3_ are the most stable phases. Mechanical properties were estimated with the calculated elastic constants, as well as the bulk modulus, shear modulus, Young’s modulus, Poisson’s ratio and Pugh’s ratio following the Voigt–Reuss–Hill scheme. As the Al content increases, the mechanical strength increases but the ductility decreases in the Ti-Al compounds. This results from the enhanced covalent bond formed by the continuously enhanced Al-*sp* hybrid orbitals and Ti-3*d* orbitals. Nb doping (~5 at.% in this study) keeps the thermodynamical and mechanical stability for the Ti-Al compounds, which exhibit slightly higher bulk modulus and better ductility. This is attributed to the fact that the Nb 4*d* orbitals locate near the Fermi level and interact with the Ti-3*d* and Al-3*p* orbitals, improving the metallic bonds based on the electronic structures.

## 1. Introduction

Ti-Al alloys possess significant potential for applications due to their high strength, stiffness, hardness, thermal stability, and corrosion resistance (e.g., in Boeing 787) [1,2,3,4]. As the aviation industry continues to advance at a rapid pace, there arises a need to further enhance the strengthening and toughening capabilities of Ti-Al alloys [5,6,7]. Considerable efforts have been dedicated to the development of techniques such as heat treatment, thermomechanical processing, and alloying to effectively control the microstructure of Ti-Al alloys [8,9,10]. Notably, the introduction of specific solute elements, such as Nb, as dopants into Ti-Al binary intermetallic compounds, has been found to induce microstructural alterations and subsequent changes in the properties.

A total of seven components and eleven configurations of Ti-Al binary intermetallic compounds have been reported based on experimental and theoretical Ti-Al binary equilibrium phase diagrams [11,12]. However, only Ti_3_Al and TiAl are presently employed as base materials for Ti-Al alloys. Other intermetallic compounds, such as TiAl_3_ with a high aluminum content and low density, have limited usage due to poor ductility and fracture toughness, despite possessing superior resistance to high-temperature oxidation and specific strength. Ghosh and Asta [13] conducted a systematic investigation on the enthalpies of formation of Ti-Al binary intermetallic compounds with varying components and configurations, classifying these compounds into stable, sub-stable, and unstable states. Jian et al. [14] studied the stability, mechanical properties and electronic structures of Ti_3_Al, TiAl, TiAl_2_, and TiAl_3_ based on the first-principles calculation, concluding that with the increase in Al content, the bulk modulus, Poisson’s ratio and ductility decrease while the shear modulus, Young’s modulus and hardness gradually increase. Tang et al. [15] performed the first-principles calculation on the long-period superstructures Al_5_Ti_2_ and Al_11_Ti_5_ to examine the elastic properties and phonon focusing on electronic structures, reporting that both compounds are mechanistically anisotropic due to strong directional bonding between the Al and Ti atoms induced by strong hybridization between Al-3*p* and Ti-3*d*.

In Ti-Al alloys, Nb primarily exists in two forms: as a constituent of the third phase, such as Ti_2_AlNb, or as a dopant incorporated into the Ti-Al binary intermetallic compound. Chen et al. [16] investigated the mechanical properties and electronic structures of Nb-doped TiAl_2_, which is a metal-stable phase with a space group of CMMM, and found that Nb doping at Al sites improved ductility more than at Ti sites. Song et al. [17] used the discrete variational cluster method to calculate the compound electronic structure and binding energy to determine the preferred occupancy of various alloying elements in γ-TiAl, reporting that Nb preferentially occupies sites in the Ti sublattice. Based on the first-principles supercell calculations of the electronic structure and total energy, Wolf et al. [18] examined the site preference of Nb atoms in the γ-TiAl and observed that Nb predominantly occupies Ti sites. Recently, Lee et al. [19] investigated the point defect formation energies of the substitutional defects based on the first-principles calculations and found Nb prefers to locate at the Ti sites instead of the Al sites; also, Nb substitution at the Ti sites increases the yield strength of the alloy. As Liu et al. [20] reviewed, in experiments, the addition of Nb in a small amount (2 at.%) can increase the ductility and fracture toughness of γ-TiAl; as the content of Nb increases to 4–10 at.%, the hot workability and creep resistance of γ-TiAl at high temperatures improves.

Up to now, most reports on the Nb doping of Ti-Al intermetallic compounds focus on TiAl_2_ and TiAl. A comprehensive study on the Nb doping of Ti-Al intermetallic compounds is still lacking [21,22,23]. In this study, we investigated the crystal structure, stability, mechanical properties, and microscopic electronic structure of Nb-doped Ti-Al intermetallic compounds, including 7 components and 11 configurations based on the first-principles calculations. It is found that Nb doping enhances the compressibility of the Ti-Al compounds under hydrostatic pressure as well as their ductility. The partial density of states show that the Nb 4*d* orbitals locate near the Fermi level and interact with the Ti-3*d* and Al-3*p* orbitals, improving the metallic bonds and accounting for improving the mechanical properties.

## 2. Materials and Methods

The periodic density functional theory (DFT) calculations were performed using the plane-wave pseudo-potential Vienna ab initio simulation package (VASP.5.4.4, Vienna, Austria) [24]. The generalized gradient approximation as formulated by Perdew, Burke and Ernzerhof (GGA-PBE) was employed for the exchange-correlation functional [25]. The projection enhanced wave (PAW) method proposed by Blöchl and implemented by Kresse and Joubert was used with a cutoff energy of 420 eV [26]. A uniform mesh grid with a spacing of 0.03 Å was used to sample the complete Brillouin zone and calculate the density of states [27]. Brillouin zone integrations were carried out with the Methfessel–Paxton technique with a 0.1 eV smearing of the electron levels [28]. The PAW pseudopotentials considered were Ti 3*p*^6^3*d*^2^4*s*^2^, Al 2*s*^2^3*p*^1^, and Nb 4*p*^6^4*d*^4^5*s*^1^. The full relaxation structure optimization method was used to obtain the ground-state crystal structure of each compound. The total energy convergence parameter during optimization was 2 × 10^−6^ eV/atom, the Hermann–Feynman force convergence parameter was 0.01 eV/Å, the tolerance shift was less than 0.002 Å, and the stress deviation per atom was less than 0.1 GPa. In addition, the single-crystal elastic matrix constants of the compounds in the ground-state structures were calculated using the stress–strain method according to the generalized Hooke’s law.

**Figure 1:**
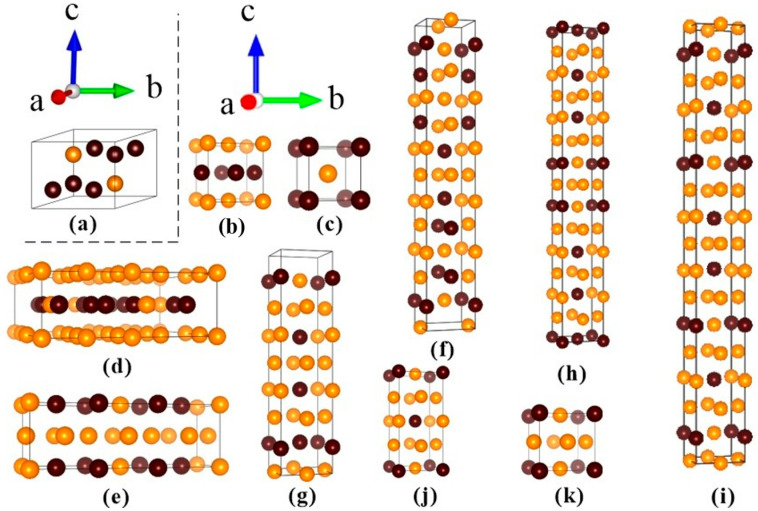
The crystal structures of the Ti-Al intermetallic compounds: (**a**) hP8-Ti_3_Al, (**b**) tP4-TiAl, (**c**) cP2-TiAl, (**d**) tP32-Ti_3_Al_5_, (**e**) tI24-TiAl_2_, (**f**) oC12-TiAl_2_, (**g**) tI16-Ti_5_Al_11_, (**h**) tP28-Ti_2_A_l5_, (**i**) tI32-TiAl_3_, (**j**) tI8-TiAl_3_, (**k**) cP4-TiAl_3_. The person symbols are written in front of the formula, and the brown and orange balls represent Ti and Al, respectively.

According to experimental and theoretical Ti-Al binary equilibrium phase diagrams [11,12], there are seven chemical compositions and 11 phases for Ti-Al intermetallic compounds, i.e., hP8-Ti_3_Al, tP4-TiAl, cP2-TiAl, tP32-Ti_3_Al_5_, tI24-TiAl_2_, oC12-TiAl_2_, tI16-Ti_5_Al_11_, tP28-Ti_2_A_l5_, tI32-TiAl_3_, tI8-TiAl_3_, and cP4-TiAl_3_, with the person symbols written in front of the formula (Figure 1). Here, cP2-TiAl, oC12-TiAl_2_, and tI32-TiAl_3_ were evaluated using thermodynamics calculations [11]. All of the structures were considered in our calculations. Furthermore, we investigated the influences of Nb doping on the mechanical properties of the Ti-Al intermetallic compounds. About 5 at.% Nb atoms were considered in order to avoid too large a change in the crystal structures.

## 3. Results and Discussion

### 3.1. Crystal Structure and Phase Stability

Table 1 lists the calculated lattice parameters and zero-temperature formation energy (Δ*H*_r_) of all Ti-Al intermetallic compounds including the experimental and other theoretical results for comparison. The formation energy was calculated using

(1) $$∆H_{r}\left({\mathrm{Ti}}_{x}{\mathrm{Al}}_{y}\right)=\frac{E_{\mathrm{tol}}\left({\mathrm{Ti}}_{x}{\mathrm{Al}}_{y}\right)\;-\;xE_{\mathrm{coh}}\left(\mathrm{Ti}\right)\;-\;yE_{\mathrm{coh}}\left(\mathrm{Al}\right)}{x+y}$$

where *E*_tol_(Ti_x_Al_y_) is the total energy of Ti_x_Al_y_ (f.u.), and *E*_coh_(Ti) and *E*_coh_(Al) are the cohesive energy of the Ti and Al crystals, respectively, which is the difference between the total energy of the Ti/Al crystal and the energy of a single Ti/Al atom [29]. As shown in Table 1, the calculated lattice parameters are quite consistent with the experimental values, with the largest difference of 2.9% for tI8-TiAl_3_. Compared to the available ${\Delta H}_{r}$ in our experiments (hP8-Ti_3_Al, tP4-TiAl, and cP4-TiAl_3_), our results are still consistent with the maximum error of 8% for hP8-Ti_3_Al. In addition, our results perfectly match other theoretical values using the same PAW-PBE method, showing only a slight difference with other theoretical methods.

Table 1 displays that the most stable structures are tP4 (P4/mmm), tI24 (I41/amd), and tI32 (I4/mmm), for the multiphase TiAl, TiAl_2_, and TiAl_3_, respectively. The cP2-TiAl is less stable than the tP4 phase with a formation energy of 13 kJ/mol higher, which is the B2 phase at high temperatures [12]. The oC12-TiAl_2_, which was reported to be metastable [39], has a formation energy 0.4 kJ/mol higher than the tI24 structure. For TiAl_3_, cP4 is the most unstable phase, with a much higher formation energy, while tI8 has a formation energy very close to the isomorphic tI32 phase. The most stable phases of tP4-TiAl, tI24-TiAl_2_, and tI32-TiAl_3_ were chosen for the subsequent calculations of the mechanical properties and Nb doping.

**Figure 2:**
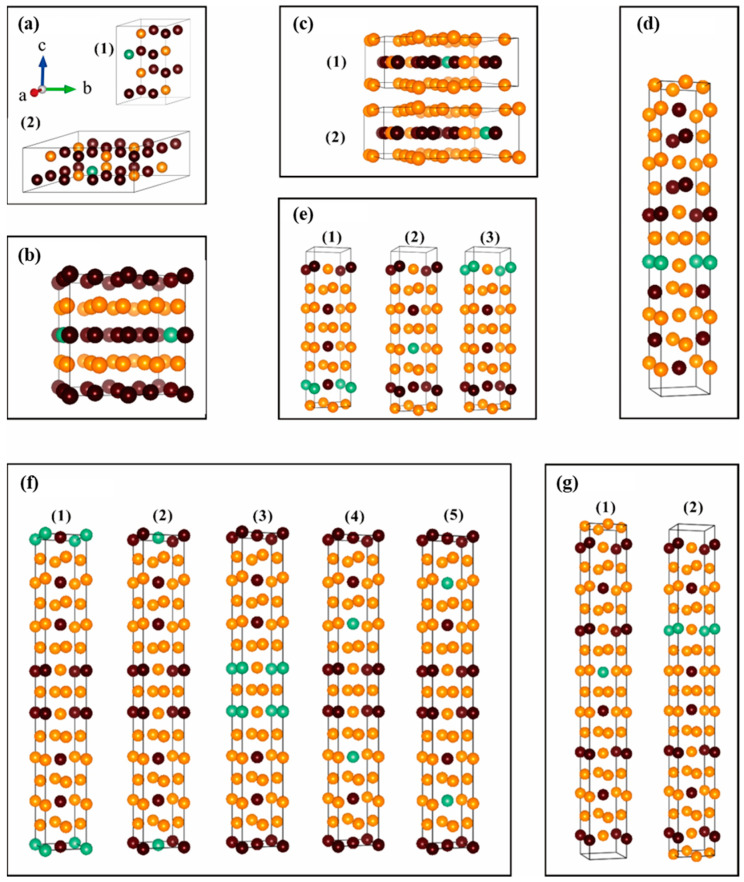
The crystal structures of the Nb-doped Ti-Al binary intermetallic compounds: (**a**) hP8-Ti_3_Al-Nb, (**b**) tP4-TiAl-Nb, (**c**) tP32-Ti_3_Al_5_-Nb, (**d**) tI24-TiAl_2_-Nb, (**e**) tI16-Ti_5_Al_11_-Nb, (**f**) tI24-Ti_2_Al_5_-Nb, and (**g**) tI32-TiAl_3_-Nb. The numbers of (1)–(5) represent the Nb doping at different crystallographic sites; the brown, orange, and green balls represent Ti, Al, and Nb, respectively.

Furthermore, we performed the Nb-doping calculations for the Ti-Al intermetallic compounds in the most stable phases, including hp8-Ti_3_Al, tP4-TiAl, tP32-Ti_3_Al_5_, tI24-TiAl_2_, tI16-Ti_5_Al_11_, tP28-Ti_2_Al_5_, and tI32-TiAl_3_. A 5% Nb atomic content was considered in order to keep the crystal structures nearly unchanged. Nb atoms were set to occupy Ti sites based on previous reports [17,18]. There were two kinds of components considered for Nb-doped hp8-Ti_3_Al, i.e., unit-cell components of Ti_11_Al_4_N_b_ (6.25 at.% Nb, 1 × 1 × 2 supercell of hp8-Ti_3_Al) and Ti_23_Al_8_N_b_ (3.125 at.% Nb, 2 × 2 × 1 supercell hp8-Ti_3_Al). For tP4-TiAl and tI24-TiAl_2_, there was only one kind of Nb doping, with the unit-cell components of Ti_15_Al_16_Nb (3.125 at.% Nb, 2 × 2 × 2 supercell of tP4-TiAl) and Ti_7_Al_16_Nb (4.167 at.% Nb), respectively. For tP32-Ti_3_Al_5_ (Ti_11_Al_20_Nb, 4.545 at.% Nb), tP28-Ti_2_Al_5_ (Ti_7_Al_20_Nb, 3.57 at.% Nb), tI16-Ti_5_Al_11_ (Ti_4_Al_11_Nb, 6.25 at.%), and tI32-TiAl_3_ (Ti_7_Al_24_Nb, 3.125 at.% Nb), there are 2, 5, 3, and 2 kinds of crystallographic sites for Nb doping, respectively, considering the coordination environment of Nb atoms and local symmetry. All of the structures are listed in Figure 2 and are named as follows: person symbol-Ti_x_Al_y_-Nb-number.

Table 2 presents the calculated lattice constants and formation energies for all the Nb-doped Ti-Al intermetallic compounds, including the percentage change in the crystal structure parameters relative to the non-doped ones. We found that ~5 at.% Nb doping does not change the crystal shape, and the volume change remains within a very small range (<0.35%). The negative formation energies indicate that the Nb-doped Ti-Al compounds are thermodynamically stable. However, in all systems, only Nb-doped TiAl_3_ (tI32-TiAl_3_-Nb-2) has lower formation energies than the non-doped ones by ~0.13 kJ/mol. The formation energy of hp8-Ti_3_Al-Nb-2 with 3.125 at.% Nb are lower than that with 6.250 at.% Nb. Clearly, Nb doping is not conducive to the thermodynamical stability of the Ti-Al intermetallic compounds. For the same component, such as tI24-Ti_2_Al_5_-Nb (3.571 at.% Nb), tI24-Ti_2_Al_5_-Nb-1 and -2 have lower formation energies, in which Nb atoms occupy Ti-rich coordinated sites. With increasing Al content, the most stable Nb-doped Ti-Al phases are hp8-Ti_3_Al-Nb-2 (3.125 at.% Nb), tP4-TiAl-Nb-1 (3.125 at.% Nb), tP32-Ti_3_Al_5_-Nb-1 (4.545 at.% Nb), tI24-TiAl_2_-Nb (4.167 at.% Nb), tI16-Ti_5_Al_11_-Nb-3 (3.571 at.% Nb), tP28-Ti_2_Al_5_-Nb-1 (3.571 at.% Nb) and tI32-TiAl_3_-Nb-2 (3.125 at.% Nb), which will be used for the following calculations of the mechanical properties.

### 3.2. Mechanical Properties

Table 3 shows the elastic matrix constants of hp8-Ti_3_Al, tP4-TiAl, tP32-Ti_3_Al_5_, tI24-TiAl_2_, tI16-Ti_5_Al_11_, tP28-Ti_2_Al_5_ and tI32-TiAl_3_, and the most stable Nb-doped phases of hp8-Ti_3_Al-Nb-2, tP4-TiAl-Nb-1, tP32-Ti_3_Al_5_-Nb-1, tI24-TiAl_2_-Nb, tI16-Ti_5_Al_11_-Nb-3, tP28-Ti_2_Al_5_-Nb-1 and tI32-TiAl_3_-Nb-2. The stiffness-related elastic constants directly reflect the mechanical stability [29], and the elastic matrix constants in Table 3 meet the mechanical stability criteria [45,46,47]. Thus, the Ti-Al compounds and Nb-doped ones are mechanically stable. The tP4-TiAl values in Table 3 are consistent with the experimental report, with a difference of ~10%. Theoretically, the elastic matrix constants are sensitive to the initial calculation parameters in the first-principles calculations, such as the cutoff energy and K-points. For tP4-TiAl, our results, calculated with a cutoff energy of 420 eV, are closer to the values from the same method (PAW-GGA) with a cutoff energy of 450 eV [48]; however, they are somewhat higher than that those with a cutoff energy of 400 eV [49]. In general, our results are consistent with previous theoretical results. As shown in Table 3, when Nb atoms are introduced into the Ti-Al intermetallic compounds, there are some changes on the elastic matrix constants: (1) hP8-Ti_3_Al-Nb-2 exhibits smaller C_11_, C_22_, C_44_, C_55_, C_66_ values, but a larger C_33_, implying enhanced anisotropy; (2) tI32-TiAl_3_-Nb-2 possess increased elastic constants, in which C_11_, C_22_ and C_33_ increase over 5 GPa; (3) for all compounds, C_33_ increases, and even more than 5 GPa for tP28-Ti_2_Al_5_-Nb-1 and tI32-TiAl_3_-Nb-2.

Based on the elastic constants, the bulk modulus (*B*), shear modulus (*G*), Young’s modulus (*E*), Poisson’s ratio (ν), and Pugh’s ratio (*K*, *B*/*G*) were calculated using the Voigt–Reuss–Hill (VRH) scheme [52,53,54]. With the calculated bulk modulus and shear modulus, Vickers hardness (*H*_v_) can be calculated according to the empirical formula proposed by Chen et al. [54]. The VRH approximation is known as the best method for the evaluation of the theoretical mechanical properties of polycrystalline materials, taking the value from the average of the Voigt and Reuss approximations [47,53]. In addition, the Debye temperature (*Θ*_D_) was evaluated in terms of the sound velocity [55,56]. All of the calculated results are shown in Table 4.

It is known that *B* reflects the compressibility of a solid under hydrostatic pressure, while *G* generally indicates the relationship between reversible deformation resistance and shear stress. *E* is defined as the ratio of stress to strain and is used to measure the stiffness of a material. A larger *E* means a higher stiffness with more covalent bond characteristics [57,58]. For Ti-Al compounds (Table 4), *B* decreases as the Al content increases, with the highest value of 116.09 GPa for Ti_3_Al and the lowest value of 106.86 GPa for TiAl_3_. *G* and *E* continually increase with increasing Al content. The Pugh’s ratio *K* (*B*/*G*) is normally used to reflect the ductility of a compound, with a critical value of 1.75, i.e., being brittle when *K* < 1.75 and ductile when *K* > 1.75 [59]. Likewise, Poisson’s ratio *ν* reflects the chemical bonding characteristics of compounds. Covalent bonds become weaker and metallic bonds become stronger as *ν* increases, with a critical value of 0.26 [59,60]. Obviously, for Ti-Al compounds, only Ti_3_Al has a *K* higher than 1.75 and *ν* larger than 0.26, showing good ductility and strong metallic bonds. As the Al content increases, *ν/K* reduces, indicating the presence of reinforcing covalent bonds. This is consistent with the results of *H*_v_ and *Θ*_D_ (Table 4), both of which increase with increasing Al content.

As shown in Table 4, all Nb-doped Ti-Al compounds possess a larger *B*, in which Ti_5_Al_11_ has the largest D-value of 5 GPa. Obviously, Nb doping can strengthen the compressibility of Ti-Al compounds under hydrostatic pressure. After Nb doping, *G* and *E* show a non-monotonic change with increasing Al content, i.e., decreasing *G* and *E* for Ti_3_Al, a very small influence on TiAl, Ti_3_Al_5_ and TiAl_2_, and 2 (*G*) and 6 (*E*) GPa increase for Ti_5_Al_11_. As the Al content further increases, this increment decreases. It can be seen that Nb doping can increase the ductility of Ti-Al compounds, which is reflected in the increased Pugh’s ratio *K* and Poisson’s ratio *ν*. Obviously, Nb doping weakens the covalent bonds and strengthens the metallic bonds; thus, the *Θ*_D_ and *H*_v_ of Nb-doped Ti-Al compounds become smaller than the non-doped ones. In addition, Nb doping has a greater influence on low-Al-content systems such as hP8-Ti_3_Al and tP4-TiAl, with the *ν* increment being about 0.01.

**Figure 3:**
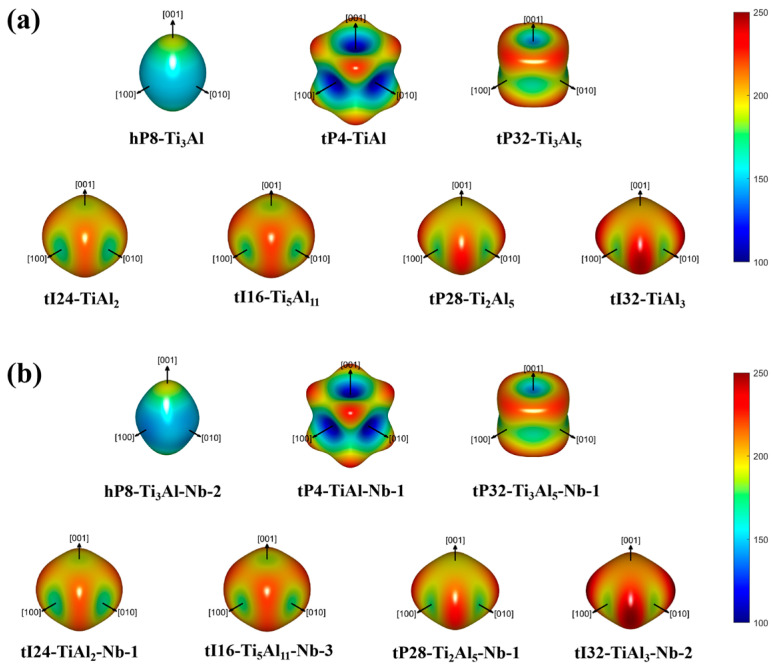
The 3D projection of the Young’s modulus of (**a**) Ti-Al and (**b**) Nb-doped Ti-Al compounds.

Figure 3a shows the three-dimensional plots of the Young’s modulus of hp8-Ti_3_Al, tP4-TiAl, tP32-Ti_3_Al_5_, tI24-TiAl_2_, tI16-Ti_5_Al_11_, tP28-Ti_2_Al_5_ and tI32-TiAl_3_. The plots of hp8-Ti_3_Al, tP4-TiAl, and tI32-TiAl_3_ are quite similar to previous theoretical reports [14]. The anisotropy of single-crystal structures usually originates from the directional properties of covalent bond. From the visual observation, hp8-Ti_3_Al seems to have a greater isotropic Young’s modulus. As the Al content increases, the Ti-Al compounds display a greater anisotropic Young’s modulus. As shown in Figure 3b, after Nb doping, the Young’s modulus anisotropy of hP8-Ti_3_Al-Nb-2 has a considerable change along the [100] and [010] directions, i.e., its anisotropy increases. However, the Young’s modulus of tI32-TiAl_3_-Nb-2 decreases slightly along the [100] and [010] directions, gently weakening its anisotropy. For other Ti-Al compounds, Nb doping has almost no influence on the anisotropy.

### 3.3. Electronic Structures

**Figure 4:**
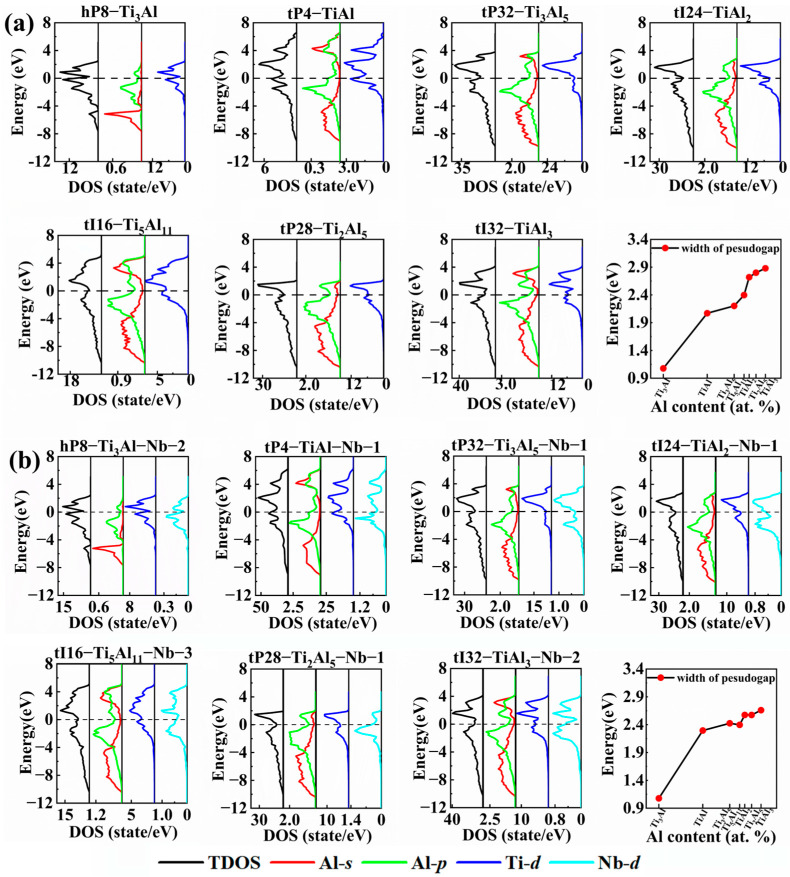
The total density of states (TDOSs), partial density of states (PDOSs) and trend of the pseudo-energy gap width with increasing Al content of the (**a**) Ti-Al and (**b**) Nb-doped Ti-Al compounds.

In order to gain an insight into the physical mechanisms, the calculations of the electronic structures were performed for the Ti-Al compounds of hp8-Ti_3_Al, tP4-TiAl, tP32-Ti_3_Al_5_, tI24-TiAl_2_, tI16-Ti_5_Al_11_, tP28-Ti_2_Al_5_ and tI32-TiAl_3_, and the most stable Nb-doped phases of hp8-Ti_3_Al-Nb-2, tP4-TiAl-Nb-1, tP32-Ti_3_Al_5_-Nb-1, tI24-TiAl_2_-Nb, tI16-Ti_5_Al_11_-Nb-3, tP28-Ti_2_Al_5_-Nb-1 and tI32-TiAl_3_-Nb-2. Figure 4 presents the total density of states (TDOSs) and the partial density of states (PDOSs). The TDOS displays a large distribution across the Fermi energy level (*E*_F_), indicating that all compounds show a metallic behavior. In addition, a pseudo-energy gap (a pronounced valley near *E*_F_) can be clearly observed from the TDOSs in Figure 4, which exists in the bonding and anti-bonding regions. The stability of a compound can be assessed based on the relative position of the *E*_F_ and the pseudo-energy gap. When the *E*_F_ lies to the right of the pseudo-energy gap, the electrons occupy the bonding region, indicating a stable structure. Conversely, when the *E*_F_ lies to the left of the pseudo-energy gap, the electrons occupy the anti-bonding region, resulting in a less stable structure. The width of the pseudo-energy gap serves as an indicator of the strength of the covalent bond, and a wider gap suggests a stronger covalent interaction [61].

As shown in Figure 4a, the pseudo-energy gap width increases with increasing Al content, implying an enhancement of the covalent bond. Thus, hP8-Ti_3_Al has the lowest pseudo-energy gap, in agreement with its best ductility and lowest Debye temperature of 496 K (Table 4). The PDOSs in Figure 4a show that the Al 3*s* and 3*p* orbitals are almost completely separated in hP8-Ti_3_Al. Near the Fermi level, Al-3*p* and Ti-3*d* form strong metallic bonds, accounting for the good ductility of hP8-Ti_3_Al. As the Al content increases, the Al 3*s* orbitals widen and hybridize with the Al-3*p* orbitals; moreover, this hybridization gradually strengthens. The enhanced Al-*sp*-hybridizing orbitals form strong covalent bonds with the Ti-3*d* orbitals, accounting for the enhancing mechanical strength and lower ductility with increasing Al content. After Nb doping, the pseudo-energy gap width reduces (Figure 4b). This indicates that Nb doping weakens the character of the covalent bond, being consistent with the results of the Poisson’s ratio and Debye temperature, as shown in Table 4. The electronic structures display that for hP8-Ti_3_Al-Nb-2 and tp4-TiAl-Nb-1, in which Al-*sp* hybridization is weak, the Nb 4*d* orbitals locate near the Fermi level (>−4 eV) and interact with the Ti-3*d* and Al-3*p* orbitals, strengthening the metallic bonds. This is consistent with the result that Nb doping increases the Poisson’s ratio *ν* more significantly for hP8-Ti_3_Al and tP4-TiAl than for other Ti-Al intermetallic compounds. As the Al content increases, although some Nb 4*d* electrons participate in the formation of covalent bonds because of the enhanced Al-*sp* hybridization, the introduction of Nb 4*d* electrons improves the metallicity of the Ti-Al compounds.

## 4. Conclusions

The first-principles density functional theory (DFT) was employed to study the crystal structures, stability, mechanical properties, anisotropy, and electronic structures of Nb-free and Nb-doped Ti-Al intermetallic compounds, including seven components and eleven crystal configurations based on the phase diagrams. The calculated total energies reveal that hP8-Ti_3_Al, tP4-TiAl, tP32-Ti_3_Al_5_, tI24-TiAl_2_, tI16-Ti_5_Al_11_, tI24-Ti_2_Al_5_, and tI32-TiAl_3_ are the most stable phases. Mechanical properties were estimated using the calculated elastic constants, as well as the bulk modulus, shear modulus, Young’s modulus, Poisson’s ratio and Pugh’s ratio following the Voigt–Reuss–Hill scheme. As the Al content increases, the bulk, shear and Young’s modulus increase but the Poisson’s ratio decreases for Ti-Al compounds, indicating the strengthened mechanical properties and weakened ductility. This is due to the enhanced covalent bonds, which are formed by the continuously enhanced Al-*sp* hybrid orbitals and Ti-3*d* orbitals. Nb doping (~5 at.% used in this study) maintains thermodynamic and mechanical stability for the Ti-Al compounds. Moreover, Nb-doped tI32-TiAl_3_ has a lower formation enthalpy than the non-doped ones. The mechanical results show that Nb doping brings a slightly larger bulk modulus and better ductility for Ti-Al compounds. The electronic structures display that the Nb 4*d* orbitals locate near the Fermi level and interact with the Ti-3*d* and Al-3*p* orbitals, strengthening the metallic bonds in the Ti-Al compounds. Nb doping also increases the mechanical anisotropy of hP8-Ti_3_Al.

## Figures and Tables

**Table 1:** The calculated lattice parameters (Å) and formation enthalpy (kJ/mol) of the Ti-Al intermetallic compounds.

	Pearson Symbol (Space Group)	Lattice Parameters and Formation Enthalpy				Method and Reference
		*a*	*b*	*c*	Δ*H*_r_	
Ti_3_Al	hP8	5.759		4.655	−27.086	PAW-GGA
	(P63/mmc)	5.759		4.655	−26.827	US-PP-GGA [14]
Ti_3_Al	hP8 (P63/mmc)	5.7372		4.6825	−27.395	US-PP-GGA [13]
		5.6496		4.5706	−28.70	FP-LMTP-LDA [30]
		5.6136		4.6649	−26.979	FLASTO-LDA [31]
		5.775		4.655		Experiment [32]
TiAl	tP4 (P4/mmm)	3.9893		4.074	−39.23	PAW-GGA
		3.994		4.079	−38.431	US-PP-GGA [14]
		3.9814		4.0803	−39.712	US-PP-GGA [13]
		3.9921		4.04	−42.00	FP-LMTP-LDA [30]
		3.9716		4.051	−42.00	FLASTO-LDA [31]
		4.001		4.071		Experiment [33]
					−40.1 ± 1	Experiment [34]
					−36.4 ± 1	Experiment [35]
					−35.1 ± 0.5	Experiment [36]
	cP2 (Pm-3m)	3.1865			−26.154	PAW-GGA
		3.1854			−25.876	US-PP-GGA [13]
		3.1529			−25.052	FLASTO-LDA [31]
Ti_3_Al_5_	tP32 (P4/mmm)	11.283		4.0305	−41.25	PAW-GGA
		11.286		4.0311	−41.640	US-PP-GGA [13]
		11.293		4.0381		Experiment [37]
TiAl_2_	tI24 (I41/amd)	3.967		24.306	−41.73	PAW-GGA
		3.9658		24.321	−42.370	US-PP-GGA [13]
		3.9628		24.068	−42.396	FLASTO-LDA [31]
		3.9711		24.313		Experiment [38]
	oC12 (Cmmm)	12.149	3.9305	4.0067	−41.346	PAW-GGA
		12.164	3.936	4.011	−40.896	US-PP-GGA [14]
		12.161	3.9322	4.0018	−42.013	US-PP-GGA [13]
Ti_5_Al_11_	tI16 (I4/mmm)	3.926		16.517	−39.519	PAW-GGA
		3.9239		16.52	−40.18	US-PP-GGA [13]
		3.923		16.519		PAW-GGA [16]
		3.917		16.524		Experiment [39]
		3.923		16.535		Experiment [40]
Ti_2_Al_5_	tP28 (P4/mmm)	3.9132		29.019	−39.808	PAW-GGA
		3.9114		29.023	−39.398	US-PP-GGA [13]
		3.912		29.004		PAW-GGA [16]
		3.905		29.196		Experiment [40]
TiAl_3_	tI32 (I4/mmm)	3.8732		33.841	−38.846	PAW-GGA
		3.875		33.84		Experiment [41]
	tI8 (I4/mmm)	3.9664		8.4797	−38.37	PAW-GGA
		3.76		8.4976	−41.44	FP-LMTO-LDA [42]
		3.799		8.5174	−39.51	FLASTO-LDA [31]
		3.8400–3.8537		8.5600–8.6140		Experiment [43]
					−36.6 ± 1.3	Experiment [44]
					−39.2 ± 1.8	Experiment [31]
	cP4 (Pm-3m)	3.9807			−35.616	PAW-GGA
		3.981			−36.583	US-PP-GGA [13]
		3.9800–4.0500			−36.907	Experiment [43]
					−36.614	Experiment [31]

**Table 2:** The calculated lattice parameters (Å) and formation enthalpy (Δ*H*_r_, kJ/mol) of the Nb-doped Ti-Al intermetallic compounds, and the percentage change in the structural parameters relative to those of the non-doped ones. *x*% is the atomic content of Nb in percentage. Boldface denotes the most stable structure in the same Ti-Al component compounds.

	*x*%	*a*	*c*	*c/a*	*V*	Δ*a*	Δ*c*	Δ(*c/a*)	Δ*V*	Δ*H*_r_
hP8-Ti_3_Al-Nb-1	6.250%	5.729	9.327	1.628	267.107	−0.327%	0.266%	1.189%	0.349%	−26.304
**hP8-Ti_3_Al-Nb-2**	**3.125%**	**11.531**	**4.658**	**0.404**	**533.271**	**0.301%**	**0.150%**	**−0.075%**	**0.171%**	**−26.784**
**tP4-TiAl-Nb-1**	**3.125%**	**7.980**	**8.157**	**1.022**	**519.509**	**0.021%**	**0.116%**	**0.095%**	**0.159%**	**−38.784**
**tP32-Ti_3_Al_5_-Nb-1**	**4.545%**	**11.297**	**4.035**	**0.357**	**514.012**	**0.124%**	**0.102%**	**−0.022%**	**0.171%**	**−40.608**
tP32-Ti_3_Al_5_-Nb-2	4.545%	11.289	4.034	0.357	514.013	0.051%	0.075%	0.024%	0.172%	−40.512
**tI24-TiAl_2_-Nb-1**	**4.167%**	**3.964**	**24.383**	**6.151**	**383.283**	**−0.069%**	**0.318%**	**0.387%**	**0.203%**	**−40.512**
tI16-Ti_5_Al_11_-Nb-1	6.250%	3.928	16.571	4.218	255.736	0.063%	0.326%	0.262%	0.452%	−38.4
tI16-Ti_5_Al_11_-Nb-2	6.250%	3.928	16.551	4.213	255.369	0.052%	0.204%	0.151%	0.307%	−37.152
**tI16-Ti_5_Al_11_-Nb-3**	**6.250%**	**3.920**	**16.582**	**4.230**	**254.817**	**−0.151%**	**0.394%**	**0.546%**	**0.090%**	**−38.592**
**tP28-Ti_2_Al_5_-Nb-1**	**3.571%**	**3.912**	**29.096**	**7.437**	**445.385**	**−0.018%**	**0.264%**	**0.282%**	**0.228%**	**−38.784**
tP28-Ti_2_Al_5_-Nb-2	3.571%	3.920	28.982	7.394	445.329	0.171%	−0.127%	−0.297%	0.215%	−37.92
tP28-Ti_2_Al_5_-Nb-3	3.571%	3.911	29.107	7.442	445.276	−0.05%	0.304%	0.355%	0.203%	−36.96
tP28-Ti_2_Al_5_-Nb-4	3.571%	3.912	29.092	7.436	445.270	−0.025%	0.252%	0.277%	0.202%	−36.96
tP28-Ti_2_Al_5_-Nb-5	3.571%	3.914	29.095	7.434	445.672	0.016%	0.261%	0.246%	0.292%	−36.48
tI32-TiAl_3_-Nb-1	3.125%	3.874	33.871	8.772	508.253	0.013%	0.414%	0.401%	0.112%	−38.208
**tI32-TiAl_3_-Nb-2**	**3.125%**	**3.871**	**33.889**	**8.754**	**507.911**	**−0.047%**	**0.141%**	**0.188%**	**0.045%**	**−38.976**

**Table 3:** The elastic constants (GPa) of the Ti-Al and Nb-doped Ti-Al intermetallic compounds.

	C_ij_ (GPa)								
	C_11_	C_12_	C_13_	C_22_	C_23_	C_33_	C_44_	C_55_	C_66_
Ti-Al compounds									
hP8-Ti_3_Al	193.9	84.1	66.5	193.9	66.5	223	63.5	63.5	54.9 ^a^
	192.2	78.2	66.8	192.2	66.8	234.2	61.6	61.6	57.0 ^b^ [14]
	202.6	67.6	78.9	202.6	78.9	202.9	61.6	61.6	67.5 ^a^ [49]
tP4-TiAl	171	88.7	85.9	171	85.9	165.5	114.1	114.1	69.8 ^a^
	168.6	88.3	80.9	168.6	80.9	174.1	111.8	111.8	73.7 ^a^ [14]
	166.4	96	88.1	166.4	88.1	179.6	119.2	119.2	76.0 ^a^ [49]
	173	83	84			168	111		75 ^a^ [48]
	186	72	74			176	101		77 ^d^ [50]
tP32-Ti_3_Al_5_	215	50	71.1	215	71.1	180.1	104.8	104.8	69.7 ^a^
	213.7	52.7	72.1			181.8	101.4		65.8 ^c^ [51]
tI24-TiAl_2_	199.2	69.5	58.4	199.2	58.4	214.6	88.5	88.5	98.7 ^a^
tI16-Ti_5_Al_11_	201.6	68.8	56.6	201.6	56.6	208.9	88.5	88.5	93.9 ^a^
	200.6	71.8	58.8			208.5	87.6		92.6 ^a^ [16]
tP28-Ti_2_Al_5_	206.1	68.1	54	206.1	54	205.5	84.5	84.5	100.2 ^a^
	218.5	62.9	48.8			221.1	102.3		117.0 ^a^ [16]
tI32-TiAl_3_	208.7	71.3	47.1	208.7	47.1	215.8	89.3	89.3	116.2 ^a^
Nb-doped Ti-Al compounds									
hP8-Ti_3_Al-Nb-2	189.9	91.3	68.2	185	66.4	226.8	60.7	62.6	53.6
tP4-TiAl-Nb-1	171.3	93.5	87.4	171.3	87.4	167.7	114.7	114.7	73.8
tP32-Ti_3_Al_5_-Nb-1	216.5	51.6	73.2	218.2	72.2	181.1	104.7	104.7	67.5
tI24-TiAl_2_-Nb-1	200.8	73	61	201.3	61.3	216.1	88.5	88.5	100.2
tI16-Ti_5_Al_11_-Nb-3	208.3	74.1	61.3	208.3	61.3	212.3	92.2	92.2	99
tP28-Ti_2_Al_5_-Nb-1	209.2	74.4	52.4	209.2	52. 4	212.7	86.2	86.2	104.6
tI32-TiAl_3_-Nb-2	214	74	47.3	214	47.3	221.2	90.6	90.6	117.8

^a^ PAW-GGA-PBE; ^b^ USPP-GGA; ^c^ PAW-GGA-PW91; ^d^ experiment.

**Table 4:** The bulk modulus *B* (GPa), shear modulus *G* (GPa), Young’s modulus *E* (GPa), Poisson’s ratio *ν*, Pugh’s ratio *K*, Vickers hardness *H*_v_, and Debye temperature *Θ*_D_ (K) of the Ti-Al and Nb-doped Ti-Al intermetallic compounds.

	*B*	*G*	*E*	*H*_v_	*ν*	*K*	*Θ*_D_
Ti-Al compounds							
hP8-Ti_3_Al	116.1	62.2	158.3	7.7	0.273	1.87	496.5
tP4-TiAl	114.3	69.1	172.5	10.2	0.240	1.65	554.7
tP32-Ti_3_Al_5_	110.5	81.4	196.1	15.2	0.204	1.36	620.0
tI24-TiAl_2_	109.5	82.7	198.2	15.9	0.198	1.32	631.7
tI16-Ti_5_Al_11_	108.5	82.2	196.9	18.2	0.197	1.32	632.8
tP28-Ti_2_Al_5_	107.7	82.7	197.4	18.4	0.194	1.30	640.2
tI32-TiAl_3_	106.9	89.7	210.3	19.5	0.172	1.19	673.1
Nb-doped Ti-Al compounds							
hP8-Ti_3_Al-Nb-2	118.2	60.4	154.8	7.0	0.282	1.96	481.8
tP4-TiAl-Nb	116.3	69.4	173.6	10.0	0.251	1.67	545.6
tP32-Ti_3_Al_5_-Nb-1	112.1	80.9	195.7	14.7	0.209	1.38	601.5
tI24-TiAl_2_-Nb	112.1	82.6	199.0	15.4	0.204	1.35	614.9
tI16-Ti_5_Al_11_-Nb-3	113.6	84.6	203.3	15.7	0.201	1.34	617.6
tP28-Ti_2_Al_5_-Nb-1	109.9	84.7	202.1	16.6	0.193	1.29	628
tI32-TiAl_3_-Nb-2	109.5	90.6	213.0	19.2	0.176	1.21	662.1

## Data Availability

Data are contained within the article.
